# ﻿*Mallotusbullatus* (Euphorbiaceae), a new species from Southwest China based on morphological characters and phylogenetic evidence

**DOI:** 10.3897/phytokeys.249.131824

**Published:** 2024-11-08

**Authors:** Jiang-Hong Yu, Zheng-Ren Chen, Ming-Tai An, Deng-Li Yu, Feng Liu, Jian Xu, Yu-Bin Tang, Yi-Ran Wang, Hua-Kai Zou

**Affiliations:** 1 College of Life Sciences, Guizhou University, Guiyang 550025, Guizhou, China; 2 Guizhou Maolan National Nature Reserve Administration, Libo 558400, Guizhou, China; 3 College of Forestry, Guizhou University, Guiyang 550025, Guizhou, China; 4 Guizhou Botanical Garden, Guiyang, 550000, Guizhou, China

**Keywords:** Euphorbiaceae, Guizhou province, karst, molecular identification

## Abstract

*Mallotusbullatus* M.T.An & J.H.Yu, **sp. nov.** (Euphorbiaceae), a species new to science discovered in Guizhou, China, is described and illustrated here, and its phylogenetic position among other *Mallotus* species is presented. Morphological, micro-morphological, and molecular evidence is presented as attestation of its novelty. The new species morphologically resembles M.philippensisvar.reticulatus and M.philippensisvar.philippensis, but it clearly differs by having bullate leaf surfaces (vs. not bullate), leaf margins entire or nearly so (vs. entire or nearly so in M.philippensisvar.philippensis and coarsely serrate in M.philippensisvar.reticulatus), leaf margins sometimes bearing red glands (vs. red glands absent), 5 sepals in staminate flowers (vs. 3–4 in M.philippensisvar.philippensis and 4 in M.philippensisvar.reticulatus), fruits with spines (vs. spines absent in M.philippensisvar.philippensis and present in M.philippensisvar.reticulatus), and abaxial leaf epidermal scattered and clustered vein hairs 0.1–0.8 mm long (vs. 0.04–0.28 mm long in M.philippensisvar.philippensis and 0.05–0.1 mm long in M.philippensisvar.reticulatus). Molecular phylogenetic analysis (BS = 100% / BS = 96%, PP = 1 / PP = 1) provides strong evidence supporting *M.bullatus* as a new species within the genus *Mallotus* and supports its placement in M.sect.Philippinenses as sister to *M.philippensis*.

## ﻿Introduction

*Mallotus* Lour. (Euphorbiaceae) is a large genus comprising approximately 150 species (Sierra et al. 2005), predominantly consisting of shrubs or trees, seldom climbers. It is mainly distributed in tropical and subtropical regions of Asia, Australia, and the Pacific, with a few species found in tropical Africa and Madagascar (Kulju et al. 2007a; Sierra et al. 2007). In China, there are approximately 30 species of *Mallotus*, mainly distributed in southern provinces and regions. The bark of some species is used for making ropes, and the seed oil is used for soap and industrial oils (Liao and Liu 1958). Additionally, *Mallotus* species are important medicinal plants and sources of dye (Kumar et al. 2006; Sharma and Varma 2011; Dhaker and Sharma 2014). The genus *Mallotus* is an important component of forest vegetation (Slik et al. 2003; Eichhorn 2006), exhibiting a variety of life history strategies. Some species act as early successional pioneers, while others are climax species. The genus occurs in a wide range of habitats at low elevations (Sierra et al. 2007).

The genus *Mallotus* was established by De Loureiro in 1790 based on *Mallotuscochinchinensis* Lour. (Loureiro 1790). In the latest taxonomic studies of Euphorbiaceae (Webster 1994; Radcliffe-Smith 2001), the genus *Mallotus* is classified in the subtribe Rottlerinae Meisn. In addition, *Mallotus* has morphological, distribution and ecological similarities with *Macaranga* Thouars, another large genus in the Euphorbiaceae. Two phylogenetic studies (Slik and van Welzen 2001; Kulju et al. 2007b) specifically investigated the relationships of *Mallotus* with related genera. Kulju et al. (2007b) identified three clades, with the majority of *Mallotus* (*Mallotus* sensu stricto [s.str.]) forming a sister group to several small genera within the *Macaranga* clade. Sierra et al. (2010) compiled various datasets including plastid (matK) and nuclear (ITS) DNA sequences, macro-morphological characteristics, and leaf anatomical data, providing a detailed analysis of the phylogeny of *Mallotus*. The study revealed that Mallotussect.Mallotus, sect. Polyadenii Pax & K.Hoffm., and sect. Stylanthus Pax & K.Hoffm. are monophyletic, while sect. Axenfeldia (Baill.) Pax & K.Hoffm. and sect. Rottleropsis Müll.Arg. are polyphyletic, and sect. Philippinenses Pax & K.Hoffm. is paraphyletic.

In 2023, during a botanical survey in the Maolan National Nature Reserve in Guizhou, China, we discovered a possibly new species of Euphorbiaceae. After more than a year of field investigations and specimen collection (GZAC-MU-0001), we conducted a field investigation on new species in Maolan National Nature Reserve, Guizhou Province, including photographing its characteristics and collecting seven live specimens. We found that its morphological characteristics resemble those of the genus *Mallotus*. To effectively distinguish the new species from other *Mallotus* species, this study utilized morphology, including pollen and leaf epidermal micromorphology, and molecular phylogenetics using ITS and matK sequences. The results led to the conclusion that the putative new species represents a new taxon.

## ﻿Materials and methods

### ﻿Morphology

Morphological features of leaves, inflorescences, flowers, and capsules were carefully observed and measured in the field, followed by detailed examination in the laboratory. Additionally, we compared specimens based on field observations and photographs taken, as well as studied related species using FAA-fixed materials and dried specimens (GZAC).

### ﻿Leaf epidermis and pollen grains

The mature, complete pollen grains and leaves collected from the field were used to measure characters through a dissecting microscope. Subsequently, they were mounted on stubs with double-sided tape, coated with a layer of gold, and then photographed using a Hitachi S-4800 scanning electron microscope. The micro-morphological characteristics of the pollen grains are described according to Wang and Wang (1983) and Nowicke and Takahashi (2002). The average size of the pollen grains is calculated based on 20 samples. The micromorphological features of the leaf epidermis of the genus *Mallotus* are stable genetic characteristics that show certain interspecific differences, reflect certain phylogenetic relationships, and can provide a basis for classification and species identification within the genus (Raju and Rao 1977; Alyas et al. 2020). Therefore, this study also investigates the leaf epidermal micromorphology of Mallotusphilippensis H.Karst., including both var. philippensis and var. reticulatus (Dunn) F.P.Metcalf. The description of leaf micromorphological features follows Fiala et al. (1994) and Živa et al. (2012), and based on seven specimens collected in the field, including the holotype and the two paratypes.

### ﻿Taxon sampling and DNA sequencing

We used a total of 36 species of *Mallotus* (Euphorbiaceae) in this study, including two individuals of the new species, and one outgroup species: *Macarangatrichocarpa* (Zoll.) Müll.Arg. We chose to use two molecular markers: ITS (ITS-1, 5.8S, and ITS-2) and matK. The ITS sequence, a highly reiterated tandem sequence in the nuclear genome, exhibits rapid changes, providing abundant variation and informative sites (Nürk et al. 2015) and a high level of species resolution accuracy (Chinese Plant Bol Group et al. 2011). The matK gene is one of the fastest-evolving genes in the chloroplast genome. It is easy to align and widely used in the study of families, genera and species (Khidir and Hongping 1997).

We extracted DNA sequences from fresh leaves of the new species and M.philippensisvar.reticulatus using a modified CTAB protocol from Doyle and Doyle (1987), followed by PCR amplification and sequencing following the protocols in referred the methods of White et al. (1990) and Taberlet et al. (1991). We downloaded DNA sequences from GenBank for the two molecular markers for the remaining species used. Taxa and GenBank accession numbers are listed in Suppl. material 1: table S1.

### ﻿Phylogenetic analysis

Sequences were aligned using default parameters in Clustal X v.1.83 (Thompson et al. 1997), followed by manual adjustments in BioEdit v.7.0 (Hall 1999). The phylogenies were constructed using Maximum Likelihood (ML) as implemented in PhyloSuite (Zhang et al. 2020) and Bayesian Inference (BI) as implemented in MrBayes v.3.0b4 (Ronquist and Huelsenbeck 2003), with the ITS and matK sequences analyzed separately. For the the nucleotide substitution model was chosen using the Akaike Information Criterion (AIC) in Modeltest v.3.06 (Posada and Crandall 1998), with the GTR+I+G model chosen for ITS and the GTR+I model for matK. For the ML analyses, the nucleotide substitution model was chosen using AIC in ModelFinder (in PhyloSuite), with the GTR+G model chosen for both regions.

## ﻿Results

### ﻿Taxonomic treatment

#### ﻿ Mallotus bullatus

Taxon classificationPlantae

M.T.An & J.H.Yu sp. nov.

0BF05FC7-A996-517F-B415-33ADD2A16BE9

urn:lsid:ipni.org:names:77351570-1

Fig. 1

##### Type.

China. • Guizhou Province, Libo County, Dawn township aquatic animals, 25°19'N, 107°56'E, alt. 700 m, 29 April 2024, *Ming-tai An, Jiang-hong Yu, Jian Xu, Feng Liu GZAC-MU-001* (holotype GZAC!).

**Figure 1.**
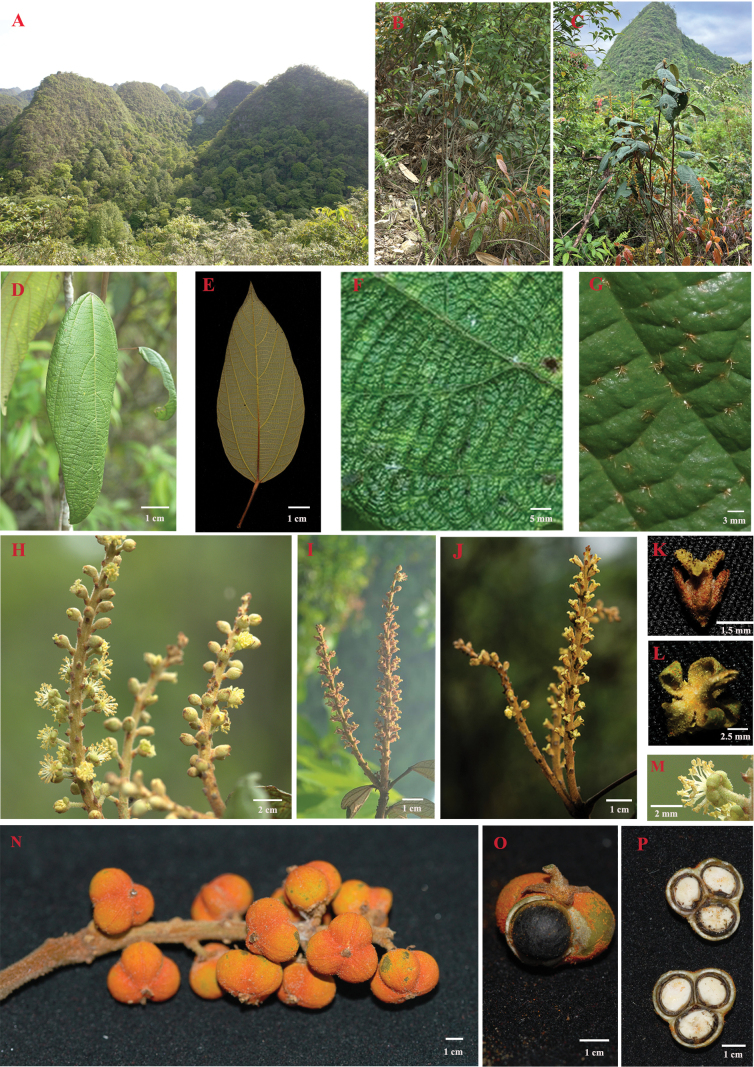
*Mallotusbullatus***A** habitat **B, C** plant **D** leaf from above **E** leaf from below **F, G** leaf lower surface **H–J** inflorescences **K** pistillate flower **L, M** staminate flower sepals **N** infructescence with mature capsules **O** capsule with part removed to show seed **P** capsule in transverse section.

##### Diagnosis.

*M.bullatus* can be distinguished from M.philippensisvar.reticulatus and M.philippensisvar.philippensis by having leaves with bullate surfaces and entire or nearly so margins, sometimes bearing red glands, 5 sepals in the staminate flowers, fruits with spines, pollen grains tricolporate with obvious furrows containing protrusions in the apertures, and abaxial leaf scattered and clustered vein hairs 0.1–0.8 mm long (Table 1).

**Table 1.** Comparison of characteristic of three species of *M.bullatus*, M.philippensisvar.philippensis, and M.philippensisvar.reticulatus.

Character	M.philippensisvar.philippensis	M.philippensisvar.reticulatus	*M.bullatus*
Habit	Small tree or shrub	Shrub	Shrub
Upper leaf midrib hairys	No	Yes	No
Leaf margins	Entire or nearly so	Coarsely serrate	Entire or nearly so
Leaf margins bearing red glands	No	No	Sometimes
Leaf surface bullate	No	No	Yes
Length of solitary or clustered hairs on leaf abaxial veins	0.04-0.28 mm (Zhang, 2018)	0.05-0.1 mm	0.1-0.8 mm
Number of staminate sepals	3-4	4	5
Pollen size	15.5(17.5)-(11.6)15.5 μm (Nowicki and Takahashi 2002)	19 × 20 μm	22 × 20 μm
Fruits with spines	No	Yes	Yes

##### Description.

***Shrubs***, 1.5–2.5 m tall; twigs, young leaves, and inflorescences densely covered with yellowish-brown disc-shaped glandular hairs. ***Leaves*** simple, alternate, ovate or lanceolate, 5–18 (-22) × 3–6 cm, thickly papery, apex acuminate, base rounded or cuneate, margins entire or nearly so, sometimes bearing red glands, surface bullate, upper surface glabrous, lower surface densely grayish-yellow clustered-tomentose, with long soft solitary or clustered hairs on the veins, and scattered red disc-like glands; basal veins 3, lateral veins 3–4 pairs, looped and joined near the margin; extrafloral nectaries, 2–4, brown, near the base; petiole round 2–5 (-9) cm long, slightly pulvinate at both ends, covered with clustered hairs. ***Inflorescences*** racemose, terminal, solitary or clustered, solely staminate or pistillate, or mixed with pistillate flowers in lower part and staminate ones in upper part; sometimes apparently bisexual flowers also present. ***Staminate inflorescences*** 5–10 cm long, bracts ovate, ca. 1 mm long, pedicel 1–2 mm long, calyx lobes 5, oblong, ca. 2 mm long, densely covered with stellate hairs, with red disc-like glands; stamens 28–30. ***Pistillate inflorescences*** s 3–8 cm long, bracts ovate, about 1 mm long; pedicels ca. 1–2 mm long; calyx lobes 4, ovate, densely covered with stellate hairs outside, ca. 3 mm long; ovary hairy, stigmas 3 split, 3–4 mm long, stigmas densely set with feather-like papillae on upper surface; some pistillate flowers sometimes bisexual, then with 1 or 2 stamens, the filaments almost as long as the anthers. ***Bisexual inflorescences*** 5–10 cm long, with 3–6 staminate flowers at the apex, lower part entirely pistillate; bracts ovate. ***Capsule*** subglobose, with spines, ca. 6–8 mm in diameter, fruit wall thickness ca.1–2 mm, 3 carpellate, densely covered with red disc-like glands; ***seeds*** black, ovate or globose, naked with late mature stage.

##### Distribution and habitat.

**Figure 2.**
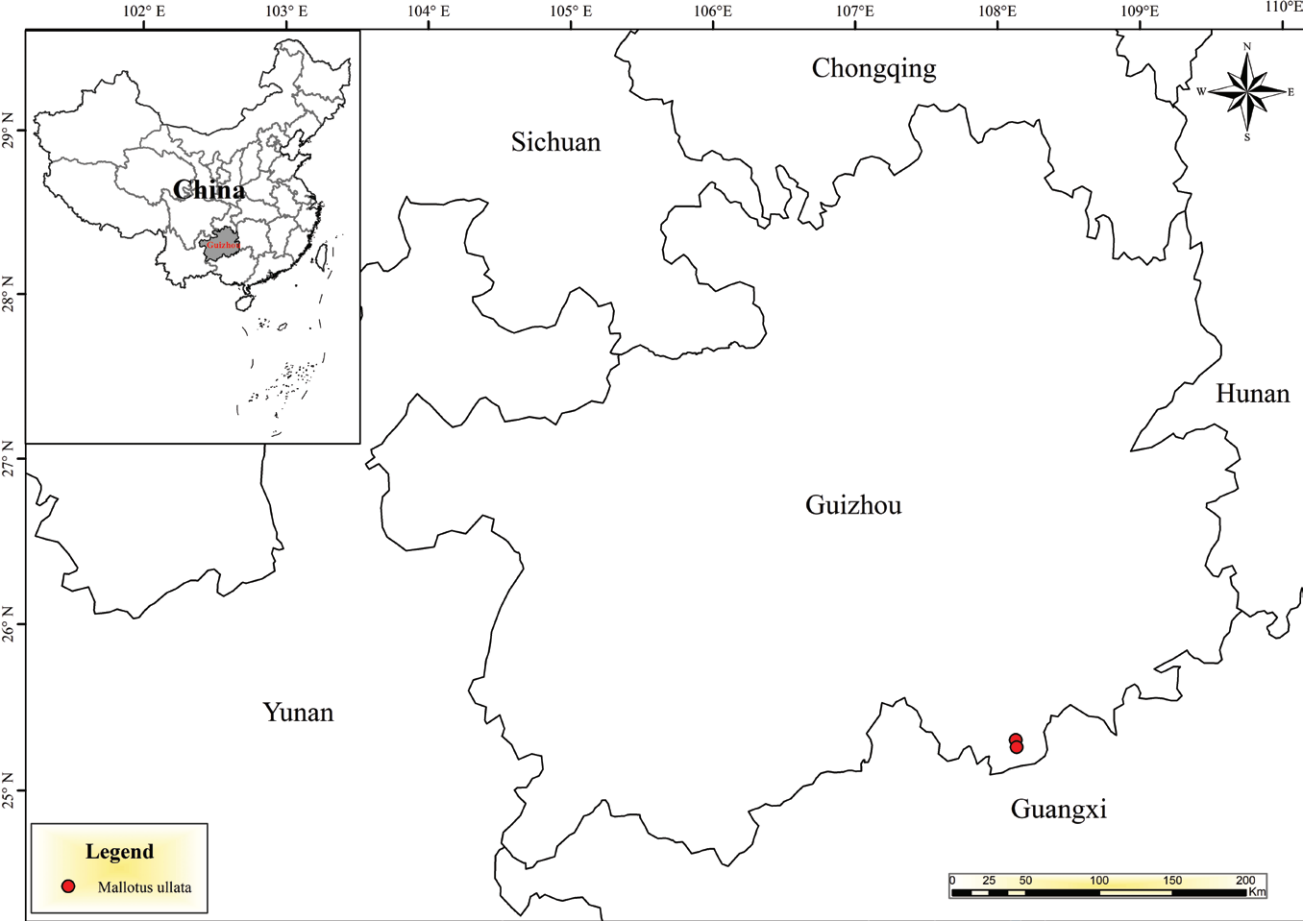
Geographical distribution of *Mallotusbullatus*.

This species is known only from the karst landscape of Libo County, Guizhou Province, China (Fig. 2), alt. 700–900 m.

##### Phenology.

Flowering from April to May, and fruiting from May to August.

##### Etymology.

“Bullatus” specifically refers to the convex leaf areoles.

##### Local name.

Simplified Chinese: 荔波野桐; Chinese Pinyin: lì bō yě tóng.

##### Leaf epidermis and palynology.

**Figure 3.**
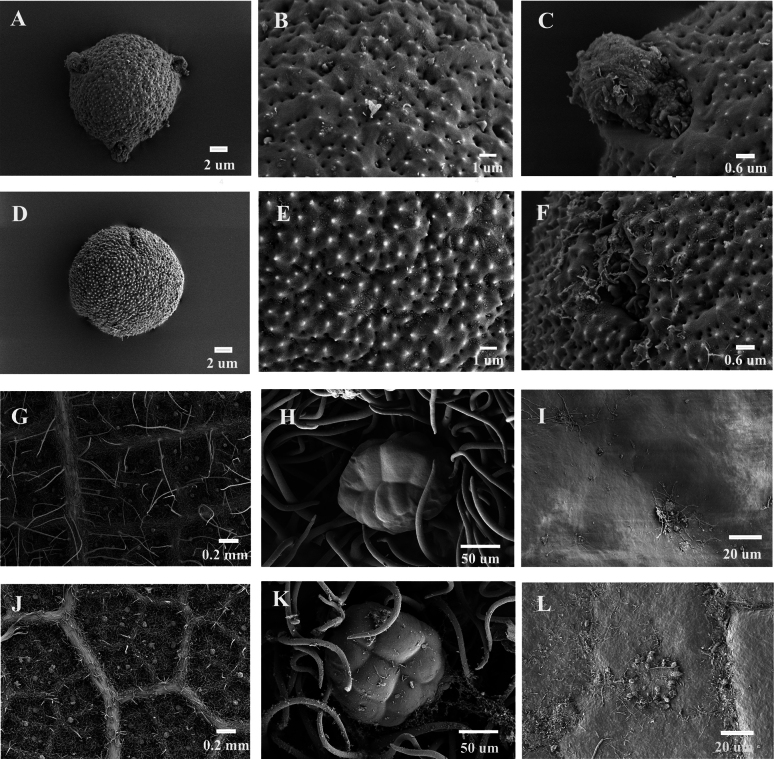
Scanning electron microscope images of *Mallotus* leaf epidermis and pollen grains **A–C** pollen grains of *M.bullatus***D–F** pollen grains of M.philippensisvar.reticulatus**G–I** lower leaf epidermis of *M.bullatus***J–L** lower leaf epidermis of *M.philippensisvar.reticulatus*.

Pollen grains of *M.bullatus* are spheroidal, with a size of 22 × 20 μm, L(long)/W(width) = 1.1, and tricolporate. They feature tricolporate furrows containing protrusions (Fig. 3A–C). Pollen grains of M.philippensisvar.reticulatus are also spheroidal, 19 × 20 μm, L/W ratio 0.95, without distinct furrows (Fig. 3D–F). The lower epidermis of *M.bullatus* leaves bears evenly distributed elliptical glands measuring 100 × 70 μm (E1 × E2: length of long equatorial axis × length of short equatorial axis), is densely covered with short clustered hairs, and has long (0.1–0.8 mm long) solitary or clustered hairs on the veins (Fig. 3G, H). Similarly, the lower epidermis of M.philippensisvar.reticulatus exhibits elliptical glands measuring 80 × 70 μm (E1 × E2), is densely covered with short clustered hairs, and has solitary or clustered hairs 0.05–0.15 mm long on the veins (Fig. 3J, K). The upper epidermis of both *M.bullatus* and M.philippensisvar.reticulatus leaves is smooth (Fig. 3I, L).

##### Conservation status.

During the period of 2023–2024, we sampled the population of *M.bullatus* and discovered two additional distribution points near the species initial discovery location (Fig. 2). Each site contained approximately 30 plants. The habitat of *M.bullatus* mainly occurs in karst scrublands, distributed from the foothills to the middle of the mountains. The plant habitat features poor soil fertility, low water retention capacity, and frequent drought conditions. Due to our current insufficient comprehensive assessment of the survival status and threats to *M.bullatus*, we cannot provide specific distribution information about this population. Therefore, we recommend categorizing *M.bullatus* as Data Deficient “DD” (IUCN 2022).

### ﻿Morphological comparisons

Morphologically, the new species is similar to *M.philippensis* in having alternate leaves, basal veins 3, and racemose inflorescences. However, the new species can be distinguished from *M.philippensis* by its bullate leaf surface (vs. not bullate), leaf margins entire or nearly so (vs. entire or nearly so in M.philippensisvar.philippensis, and coarsely serrate in M.philippensisvar.reticulatus), leaf margins sometimes bearing red glands (vs. not red glands), fruits with spines (vs. absent in M.philippensisvar.philippensis and present in M.philippensisvar.reticulatus), 5 sepals in staminate flower (vs. 3–4 in M.philippensisvar.philippensis and 4 in M.philippensisvar.reticulatus) (Table 1, Suppl. material 1: fig. S1).

### ﻿Phylogenetic position

#### ﻿Nuclear data phylogenetic analyses

**Figure 4.**
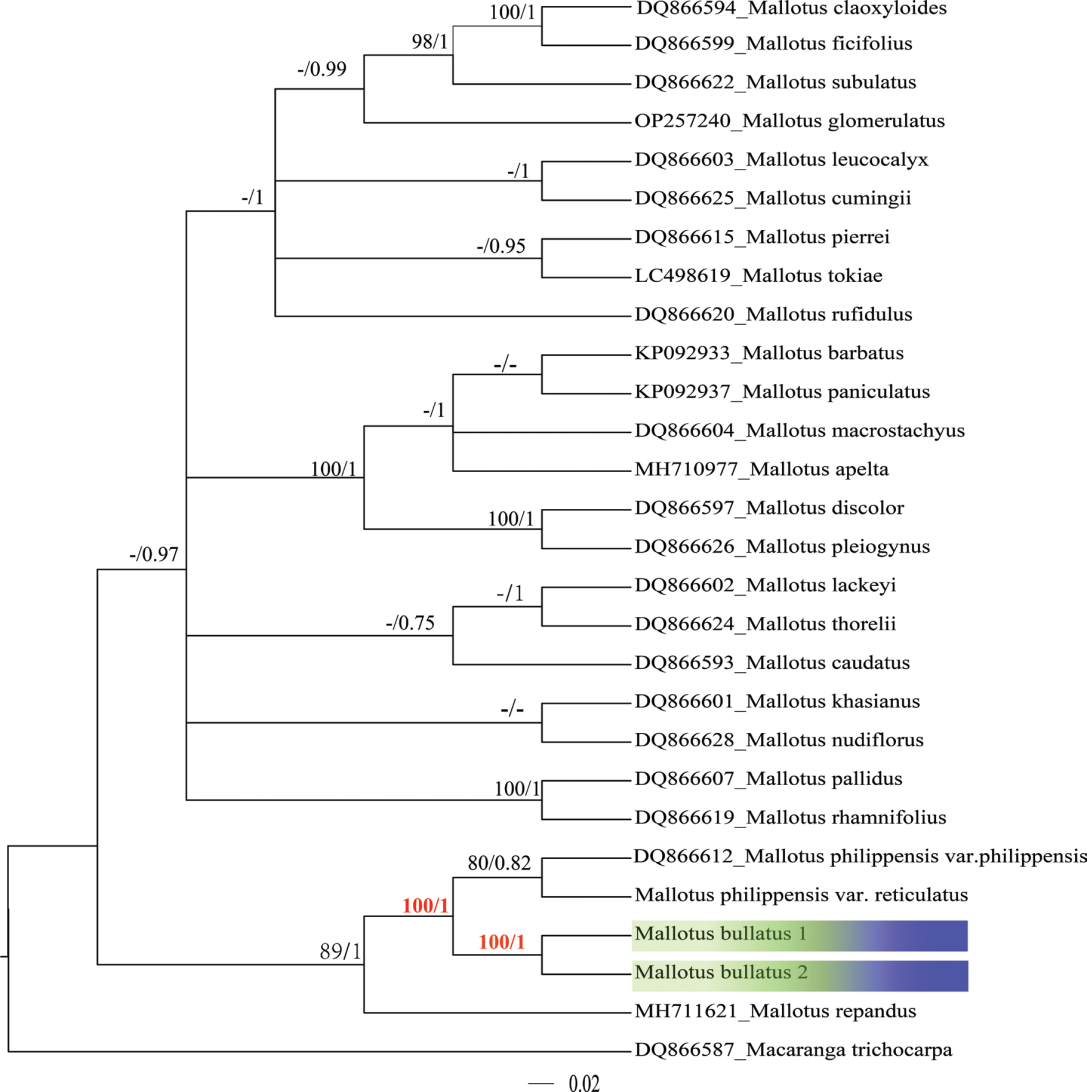
Partial Bayesian consensus phylogram based on ITS sequences. Numbers above branches are Bayesian posterior probabilities (PP) and Bootstrap probabilities (BS) (only PP values > 0.70, BS > 80 shown).

The length of the aligned ITS sequences of *M.bullatus* is 760 bp. Based on a dataset of 28 ITS sequences with 182 informative loci, phylogenetic relationships were analyzed using both Bayesian Inference (BI) and Maximum Likelihood (ML) methods (Fig. 4). The two sequences from the new species both originate from the same population, forming a strongly supported monophyletic clade (Fig. 4: BS = 100%, PP = 1). Mallotusphilippensisvar.philippensis and M.philippensisvar.reticulatus are sister taxa with strong support (Fig. 4: BS = 100%, PP = 1), and they form a strongly supported sister group relationship with *M.bullatus* (Fig. 4: BS = 98%, PP = 1).

#### ﻿Plastid data phylogenetic analysis

**Figure 5.**
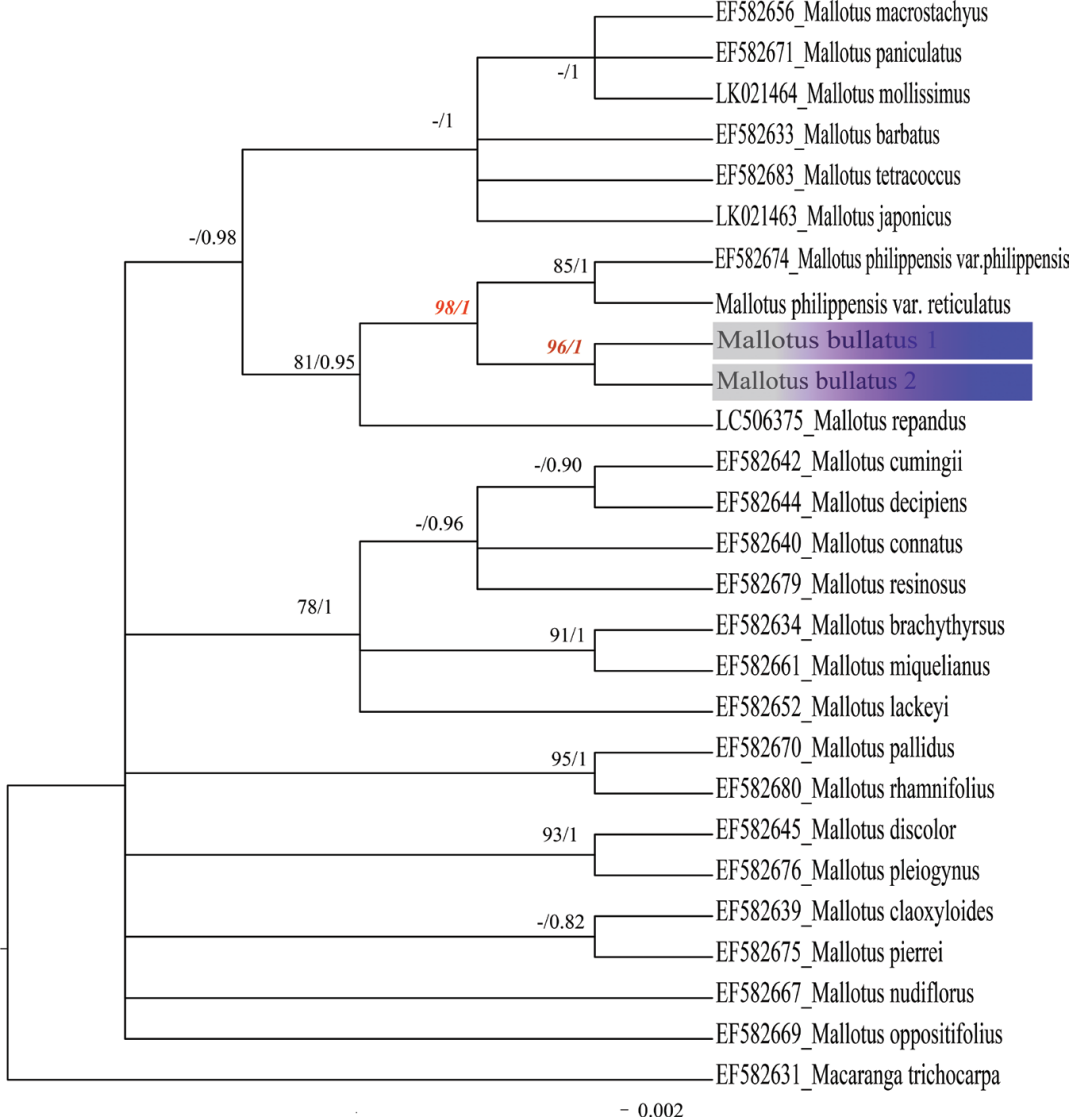
Partial Bayesian consensus phylogram based on matK sequences. Numbers above branches are Bayesian posterior probabilities (PP) and Bootstrap probabilities (BS) (only PP values > 0.70, BS > 80 shown).

The aligned matK sequences of *M.bullatus* are 2000 bp in length. Based on a dataset of 27 matK sequences with 557 informative loci, both Bayesian Inference (BI) and Maximum Likelihood (ML) analyses indicate that the two sequences from the new species form a strongly supported monophyletic clade (Fig. 5: BS = 96%, PP = 1). Mallotusphilippensisvar.philippensis and M.philippensisvar.reticulatus forms a weakly supported sister clade (Fig. 5: BS = 80%, PP = 0.82), and they form a strongly supported sister group relationship with *M.bullatus* (Fig. 5: BS = 100%, PP = 1).

## ﻿Discussion

According to the classification in Sierra et al. (2010), *M.bullatus* belongs to sect. Philippinenses, characterized by shrubby habit, alternate leaves, 3 basal leaf veins, and capsules densely covered with orange-red glands. Both phylogenetic trees (ITS and matK; Figs 4, 5) indicate that *M.bullatus* is a distinct member of the genus, and furthermore, support its sister group relationship with M.philippensisvar.philippensis plus, M.philippensisvar.reticulatus; these three species form a weakly supported clade with *M.repandus* (Figs 4, 5), also in sect. Philippinensis, thus corroborating the evidence provided by the morphological and micro-morphological observations. The two known populations of *M.bullatus* show no consistent morphological differences. Although *M.bullatus* forms a clade with M.philippensisvar.philippensis and M.philippensisvar.reticulatus, it differs from both taxa in its bullate leaf surfaces, length of hairs on the leaf abaxial veins, and number of sepals in the staminate flowers (Table 1).

### ﻿Additional specimen examined

Mallotusphilippensisvar.reticulatus (Dunn) F. P. Metcalf. —China. Fujian: Collected on Mr. Dunn’s expedition to Central, China. Apr. to Jun., 1905, 3429 (HH); West lake, Chenxiang town, Changtai district, Zhangzhou city. Jun. 11, 1976, *Wang QJ*, 012320 (AU). Jangxi: Yangling, Chongyi County, Ganzhou City, 24°29'N, 103°54'E alt. 1092 m, May 15, 2024, *Yu JH*, *Tang YB*, *Wang YR*, 20240501 (GZAC). M.philippensisvar.philippensis (Lam.) Müll. Arg. — Yunan: roadside at the edge of forests, alt. 800 m. 2000, *Shui YM*, *Chen WH* 13773 (PE). Sichuan: Jinjia Village, Sutie National Nature Reserve, Panzhihua, Sichuan Province, 26°37'29.7"N, 101°33'03.1"E, alt. 1653 m. Sept. 16, 2010. *Yang Y*, *Huang JH*, *Yang YQ*, *Liu B*, *Ye JF* 551 (PE). **Paratypes.***Mallotusbullatus* — Guizhou: Rao gu village, Dawn township aquatic animals, Libo County, 25°19'N, 107°56'E, alt. 800 m, Apr. 29, 2024, *An MT*, *Yu JH*, *Xu J*, *Liu F* 202306-1 (GZAC); La nei village, Libo County, 28°21'N, 107°56'E, alt. 750 m, Apr. 30, 2024, *An MT*, *Yu JH*, *Xu J*, *Liu F* 202306-2 (GZAC). **Other specimens.***Mallotusbullatus* — Guizhou: Yiba Mountain, Lane Village, Dawn township aquatic animals, Libo County, 25°16'N, 107°55'E, alt. 950 m, Jul. 26, 2024, *Yu JH*, *Tang YB*, *Liu F* 001 (GZAC); Yiba Mountain, Lane Village, Dawn township aquatic animals, Libo County, 25°16'N, 107°55'E, alt. 780 m, Jul. 28, 2024, *Yu JH*, *Tang YB*, *Liu F* 002 (GZAC); Dawn township aquatic animals, Libo County, 25°16'N, 107°57'E, alt. 840 m, Jul. 22, 2024, *Yu JH*, *Tang YB*, *Liu F* 003 (GZAC); Dawn township aquatic animals, Libo County, 25°16'N, 107°57'E, alt. 700 m, Jul. 22, 2024, *Yu JH*, *Tang YB*, *Liu F* 004 (GZAC).

## Supplementary Material

XML Treatment for Mallotus bullatus

## ﻿Additional information

### Author ORCIDs

Jiang-Hong Yu https://orcid.org/0000-0003-1765-8557

Ming-Tai An https://orcid.org/0000-0003-3886-0287

Deng-Li Yu https://orcid.org/0009-0004-4577-6678

Feng Liu https://orcid.org/0009-0003-7031-4494

Jian Xu https://orcid.org/0000-0002-0714-0917

Yu-Bin Tang https://orcid.org/0009-0007-8460-2242

Yi-Ran Wang https://orcid.org/0009-0008-6030-8601

Hua-Kai Zou https://orcid.org/0009-0009-2029-6068

### Data availability

All of the data that support the findings of this study are available in the main text or Supplementary Information.

## Supplementary materials

Supplementary material 1 Additional information Data type docx Explanation note **table S1.** Taxa and GenBank accession numbers for thesequences used in this study. **fig. S1.**Mallotusphilippensisvar.philippensis (Lam.) Müll.Arg. and M.philippensisvar.reticulatus (Dunn) F.P.Metcalf. **A, B** leaf of Mallotusphilippensisvar.reticulatus**C** holotype of Mallotusphilippensisvar.philippensis**D** holotype of Mallotusphilippensisvar.reticulatus**E** merotype of Mallotusphilippensisvar.reticulatus. https://binary.pensoft.net/file/1170440This dataset is made available under the Open Database License (http://opendatacommons.org/licenses/odbl/1.0/). The Open Database License (ODbL) is a license agreement intended to allow users to freely share, modify, and use this Dataset while maintaining this same freedom for others, provided that the original source and author(s) are credited.Jiang-Hong Yu, Zheng-Ren Chen, Ming-Tai An, Deng-Li Yu, Feng Liu, Jian Xu, Yu-Bin Tang, Yi-Ran Wang, Hua-Kai Zou
