# Supplementary material for: ﻿Mallotusbullatus (Euphorbiaceae), a new species from Southwest China based on morphological characters and phylogenetic evidence
Source: PhytoKeys. 2024 Nov 8;249:13–25. doi: 10.3897/phytokeys.249.131824 (PMC11568409; doi:10.3897/phytokeys.249.131824)
Supplement: Supplementary material 1 — Additional information [file phytokeys-249-013_article-131824__-s001.docx]

**Supporting Information**

**Table S1.** Species and GenBank accession numbers for the marker sequences used in this study.

| Species | GenBank Accession number | |
| --- | --- | --- |
|  | ITS | matK |
| *Macaranga trichocarpa* (Zoll.) Müll.Arg. | DQ866587 | EF582631 |
| *Mallotus apelta* (Lour.) Müll.Arg. | MH710977 |  |
| *Mallotus barbatus* (Wall. ex Baill.) Müll.Arg. | KP092933 | EF582633 |
| *Mallotus brachythyrsus* Merr. |  | EF582634 |
| *Mallotus caudatus* Merr. | DQ866593 |  |
| *Mallotus claoxyloides* (F.Muell.) Müll.Arg. | DQ866594 | EF582639 |
| *Mallotus conatus* M.Aparicio |  | EF582640 |
| *Mallotus cumingii* Müll.Arg. | DQ866625 | EF582642 |
| *Mallotus decipiens* Müll.Arg. |  | EF582644 |
| *Mallotus discolor* F.Muell. ex Benth. | DQ866597 | EF582645 |
| *Mallotus ficifolius* (Baill.) Pax & K.Hoffm. | DQ866599 |  |
| *Mallotus glomerulatus* Welzen | OP257240 |  |
| *Mallotus japonicus* (L.f.) Müll.Arg. |  | LK021463 |
| *Mallotus khasianus* Hook.f. | DQ866601 |  |
| *Mallotus lackey* Elmer | DQ866602 | EF582652 |
| *Mallotus leucocalyx* Müll.Arg. | DQ866603 |  |
| *Mallotus macrostachyus* (Miq.) Müll.Arg. | DQ866604 | EF582656 |
| *Mallotus miquelianus* (Scheff.) Boerl. |  | EF582661 |
| *Mallotus mollissimus* (Geiseler) Airy Shaw |  | LK021464 |
| *Mallotus nudiflorus* (L.) Kulju & Welzen | DQ866628 | EF582667 |
| *Mallotus oppositifolius* (Geiseler) Müll.Arg. |  | EF582669 |
| *Mallotus pallidus* auct. non (Airy Shaw) Airy Shaw | DQ866607 | EF582670 |
| *Mallotus paniculatus* (Lam.) Müll.Arg. | KP092937 | EF582671 |
| *Mallotus philippensis* (Lam.) Müll.Arg. var. *philippensis* | DQ866612 | EF582674 |
| *Mallotus pierrei* (Gagnep.) Airy Shaw | DQ866615 | EF582675 |
| *Mallotus pleiogynus* Pax & K.Hoffm. | DQ866626 | EF582676 |
| *Mallotus repandus* (Willd.) Müll.Arg. | MH711621 | LC506375 |
| *Mallotus resinosus* (Blanco) Merr. |  | EF582679 |
| *Mallotus rhamnifolius* (Willd.) Müll.Arg. | DQ866619 | EF582680 |
| *Mallotus rufidulus* (Miq.) Müll.Arg. | DQ866620 |  |
| *Mallotus subulatus* Müll.Arg. | DQ866622 |  |
| *Mallotus tetracoccus* (Roxb.) Kurz |  | EF582683 |
| *Mallotus thorelii* Gagnep. | DQ866624 |  |
| *Mallotus tokiae* Welzen | LC498619 |  |
| *Mallotus bullatus* 1 | PQ227170 | PQ384949 |
| *Mallotus bullatus* 2 | PQ227171 | PQ384950 |
| *Mallotus philippensis var. reticulatus* (Dunn) F.P.Metcalf | PQ227172 | PQ468955 |

**
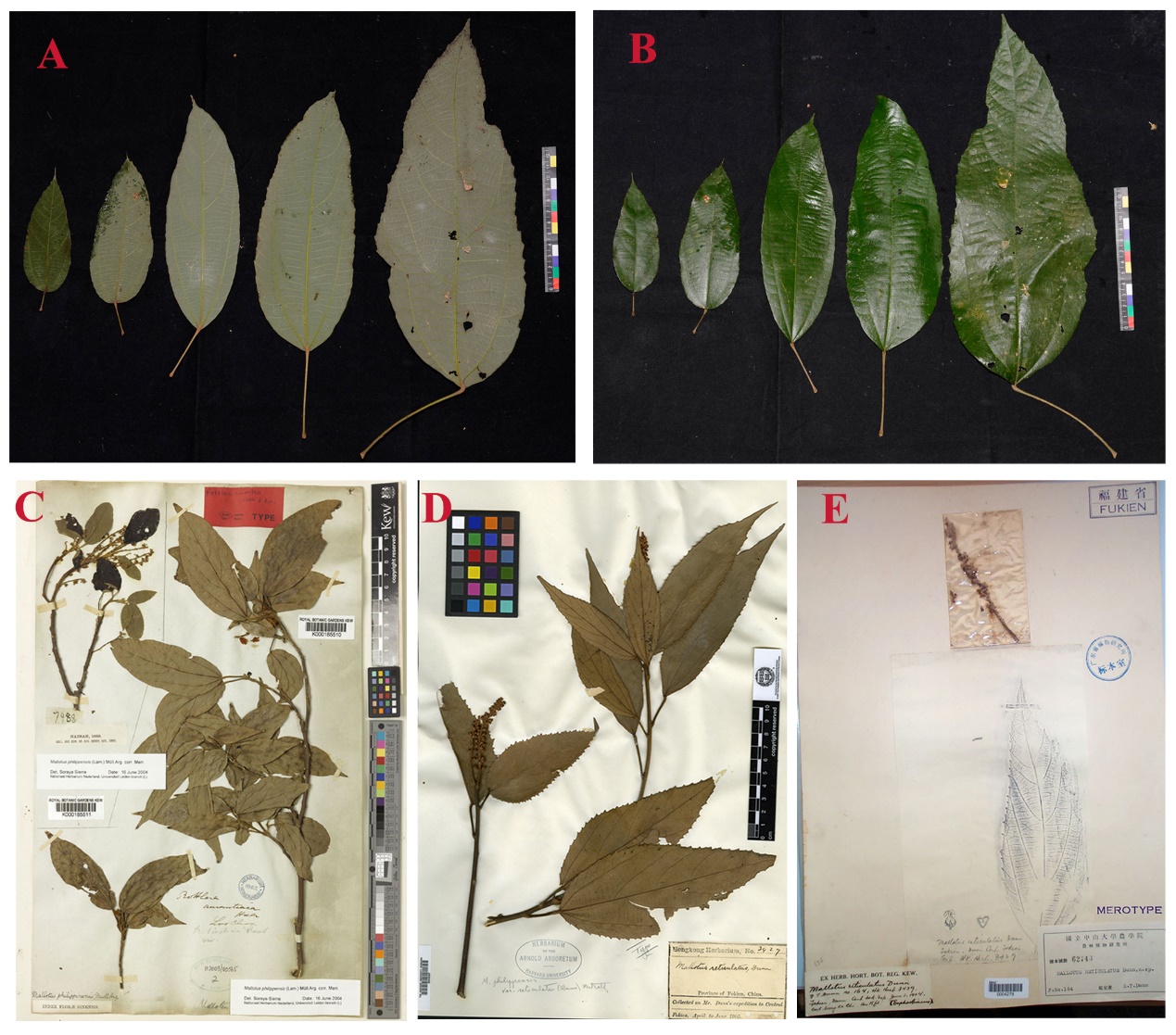
**

**Figure S1** *Mallotus philippensis* (Lam.) Müll.Arg. var. *philippensis* and *M. philippensis* var. *reticulatus* (Dunn) F.P.Metcalf. **A-B** leaf of *Mallotus philippensis* var. *reticulatus* **C** holotype of *Mallotus* *philippensis* **D** holotype of *Mallotus philippensis* var. *reticulatus* **E** merotype of *Mallotus philippensis* var. *reticulatus*
